# Supplementary material for: Effect of UV Filters during the Application of Pulsed Light to Reduce Lactobacillus brevis Contamination and 3-Methylbut-2-ene-1-thiol Formation While Preserving the Physicochemical Attributes of Blonde Ale and Centennial Red Ale Beers
Source: Foods. 2023 Feb 4;12(4):684. doi: 10.3390/foods12040684 (PMC9955924; doi:10.3390/foods12040684)
Supplement: Supplementary file 1 [file foods-12-00684-s001.zip › foods-2160633-supplementary.pdf]

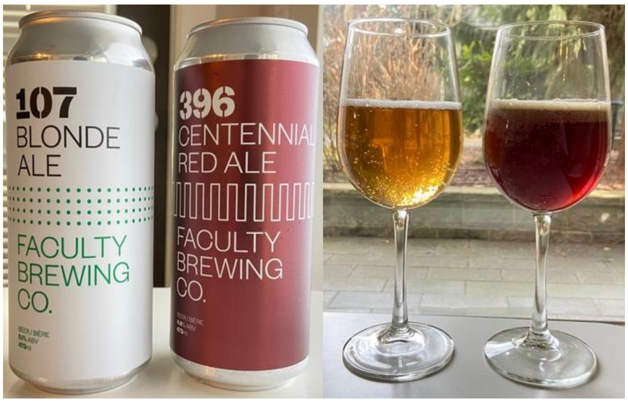

Supplementary Figure S1. Left to right: Canned blonde ale, canned centennial red ale, poured blonde ale (light), poured centennial red ale (dark)

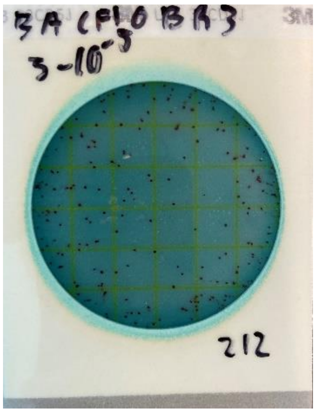

Supplemetary Figure S2. *L. brevis* colonies in Petrifilms after incubation.

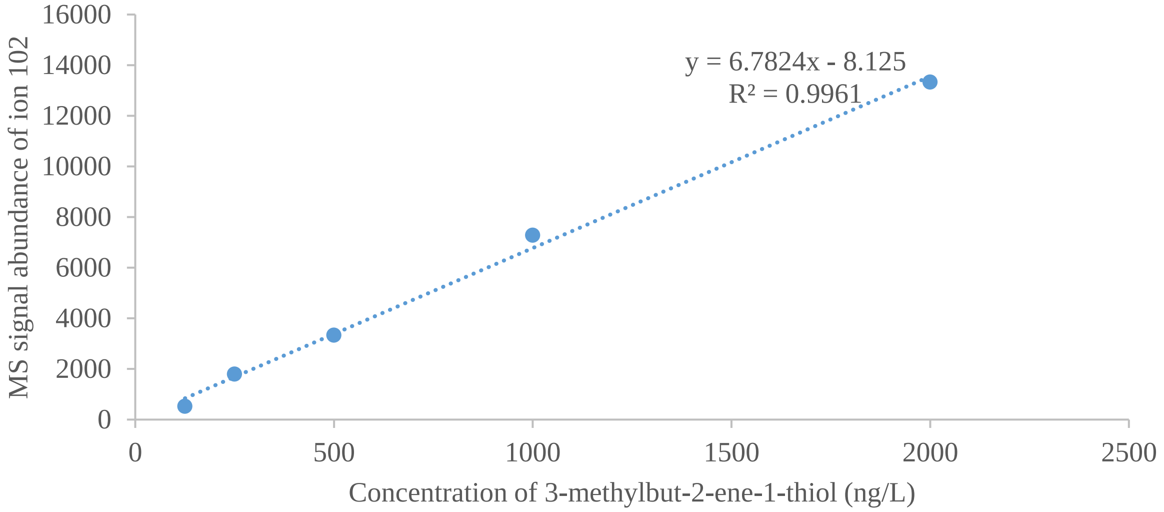

.

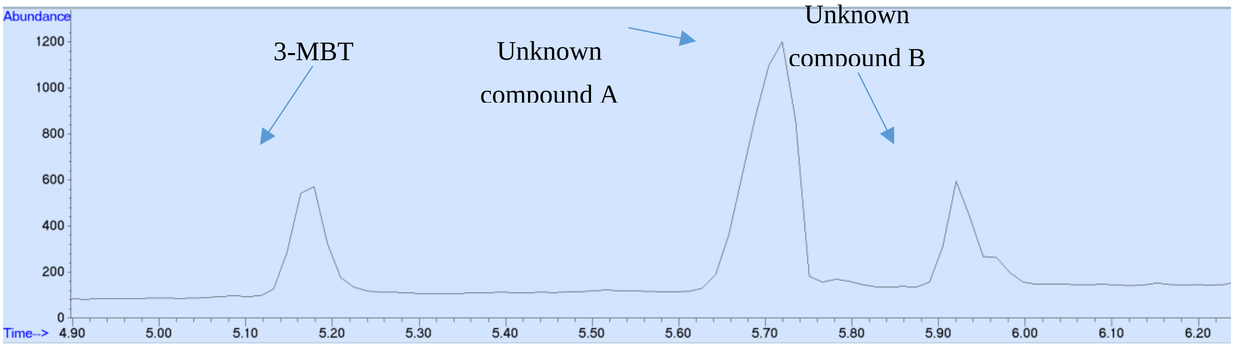

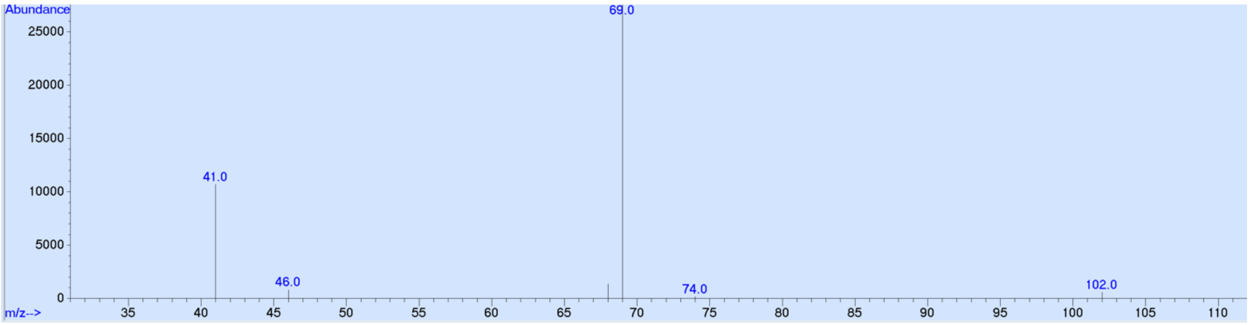

Supplementary Figure S3 (**a**) Calibration curve for 3-methylbut-2-ene-1-thiol based on the abundance of ion 102. (**b**) GC chromatogram of BA beer treated without filter for 0.5 min (Time was expressed in min. 3-MBT: 3-methylbut-2-ene). (**c**) Electron impact mass spectrum, in SIM mode, of the 3-methylbut-2-ene-1-thiol peak of the BA beer sample treated without filter for 0.5 min.

Supplementary Table S1. Microbrewery-published ingredient list for 10 gallons of finished product

Ingredient class	Blonde ale	Centennial red ale
Malt	5.7 kg Canadian 2-Row	5.0 kg Canadian 2-Row
	1.1 kg Vienna Malt	0.9 kg Simpsons Double Roasted Crystal (DRC)
	1.1 kg Carafoam	0.9 kg Carafoam
	1.1 kg Wheat Malt	
Hops	0.02 kg Magnum	0.06 kg Centennial
Yeast	1 package (11.5 g) Safale S-04	1 package (11.5 g) Safale US-05

Supplementary Table S2. Microbrewery-published brewing process details

Process	Blonde ale	Centennial red ale
Mash	60 minutes at 66°C	60 minutes at 66°C
Hops schedule (time before wort flame-out)	0.02 kg Magnum - boil 60 minutes	0.02 kg Centennial - boil 60 minutes<br>0.02 kg Centennial - boil 15 minutes<br>0.02 kg Centennial - boil 1 minute
Fermentation	14-21 days at 21°C	14-21 days at 21°C
